# Hydrophobic moment drives penetration of bacterial membranes by transmembrane peptides

**DOI:** 10.1016/j.jbc.2023.105266

**Published:** 2023-09-19

**Authors:** Tyler S. Johnson, Aleksandra A. Bourdine, Charles M. Deber

**Affiliations:** 1 Program in Molecular Medicine, Research Institute, The Hospital for Sick Children, Toronto, Ontario, Canada; 2 Department of Biochemistry, University of Toronto, Toronto, Ontario, Canada

**Keywords:** peptides, transmembrane peptides, hydrophobic moment, gram-negative bacteria, bacterial membrane, membrane biophysics, lipid bilayer, efflux pump inhibitor, protein-protein interaction

## Abstract

With antimicrobial resistance (AMR) remaining a persistent and growing threat to human health worldwide, membrane-active peptides are gaining traction as an alternative strategy to overcome the issue. Membrane-embedded multi-drug resistant (MDR) efflux pumps are a prime target for membrane-active peptides, as they are a well-established contributor to clinically relevant AMR infections. Here, we describe a series of transmembrane peptides (TMs) to target the oligomerization motif of the AcrB component of the AcrAB-TolC MDR efflux pump from *Escherichia coli*. These peptides contain an N-terminal acetyl-A-(Sar)_3_ (sarcosine; N-methylglycine) tag and a C-terminal lysine tag—a design strategy our lab has utilized to improve the solubility and specificity of targeting for TMs previously. While these peptides have proven useful in preventing AcrB-mediated substrate efflux, the mechanisms by which these peptides associate with and penetrate the bacterial membrane remained unknown. In this study, we have shown peptide hydrophobic moment (μH)—the measure of concentrated hydrophobicity on one face of a lipopathic α-helix—drives bacterial membrane permeabilization and depolarization, likely through lateral-phase separation of negatively-charged POPG lipids and the disruption of lipid packing. Our results show peptide μH is an important consideration when designing membrane-active peptides and may be the determining factor in whether a TM will function in a permeabilizing or non-permeabilizing manner when embedded in the bacterial membrane.

Antimicrobial resistance (AMR) levels have grown exponentially over the last decade and are estimated to contribute to upwards of 10 million deaths per year (1). With low estimates in the range of 700,000 deaths annually, a considerable body of literature has been dedicated to developing new strategies to overcome this persistent issue (2). Among a variety of mechanisms by which bacteria can develop resistance to antimicrobials, the expression of multidrug-resistant (MDR) efflux pumps has been recognized as playing an early and prominent role in a bacterium’s path toward multidrug resistance (3, 4). In the case of Gram-negative species of bacteria, the resistance-nodulation-division family of MDR efflux pumps is the major contributors to clinically relevant AMR infections, including the well-characterized AcrAB-TolC efflux system from *Escherichia coli* (5, 6). Substrate extrusion is conferred by the inner membrane-embedded AcrB component of the tripartite complex, which functions as an obligate trimer to efflux a wide range of antimicrobials, including dyes, biocides, and nearly every class of antibiotic (7, 8). AcrB oligomerization is dependent on an interaction between transmembrane helices 1 (TM1) and 8 (TM8), the mutagenic disruption of which abolishes the efflux activity of the entire AcrAB-TolC complex (9) (Fig. 1). While this interface appears a promising target for treating Gram-negative bacterial infections, there are currently no efflux pump inhibitors (EPIs) approved for use in the clinic (10).

**Figure 1:**
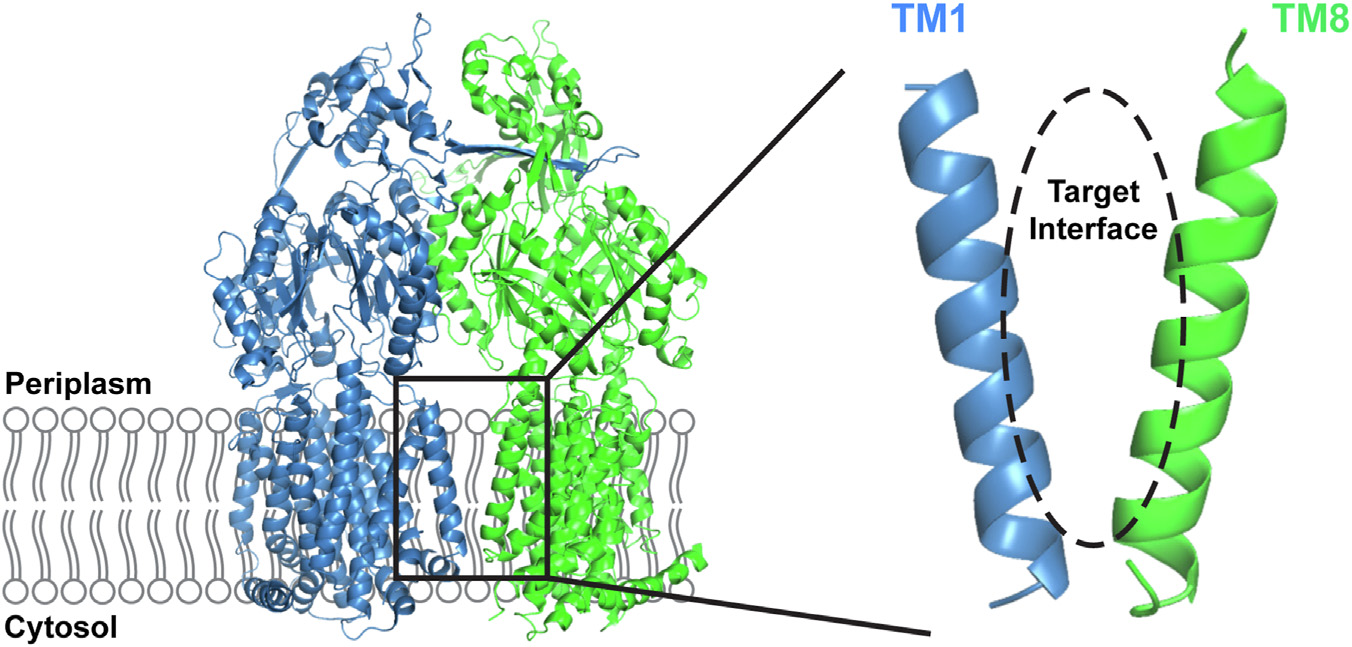
**Structure of the AcrB component of the AcrAB-TolC efflux protein from *E. coli*.** Chains A and B of AcrB are shown in *blue* and *green*, respectively. Chain C has been removed for ease of visualization. AcrB oligomerization is mediated in part by an interaction between TM1 and TM8, highlighted in the *black box* and expanded on the right. TM1-based inhibitors are designed to interfere with the target interface highlighted by the *black dashed oval*. Structure adapted from PDB: 2DHH (7).

Targeting efflux pumps for the treatment of clinically relevant bacterial infections remains a relatively novel effort and has been recognized as a potential “antibiotic resistance breaker” strategy (2, 11). To target these efflux pumps, researchers have considered membrane-active peptides, which can be categorized as antimicrobial peptides (AMPs), cell-penetrating peptides (CPPs) (12), or transmembrane peptides (TMs) (13, 14). AMPs rely on electrostatic interactions between cationic peptides and anionic bacterial membranes to induce bacterial cell death via direct membrane disruption in pore-dependent and -independent mechanisms, whereas CPPs function to cross the bacterial membrane to act on an intracellular target (15, 16). TMs are a relatively understudied class of membrane-active peptides designed to target protein-protein interactions (PPIs) that have shown promise in the fields of cancer and neurodegeneration research (17, 18).

To date, TM1-based peptides have proven useful in preventing AcrB-mediated efflux that results in adjuvant activity when combined with antimicrobials (19). Despite these successes, the distinct mechanisms by which TMs associate with and penetrate the bacterial membrane, as well as the impacts of peptide insertion on membrane stability, have not been thoroughly explored. One such mechanism may involve peptide hydrophobic moment (μH), which has previously been noted in the context of AMPs, where the peptide μH acts as a measure of hydrophobicity for the non-polar face of an amphipathic α-helix, generally dictating the orientation with which the peptide will associate with the lipid bilayer (20). By contrast, in the case of TMs, a polar face of the α-helix is not necessarily present: instead, we can define two hydrophobic faces, with one face having a concentration of relatively greater hydrophobic character than the other. We have coined this latter helical patterning as a “lipopathic” sequence, which evokes the situation where both faces of the α-helix have the potential to associate – but to different extents—with the lipid bilayer (21). In the present study, we uncover the role the μH plays in a peptide’s ability to penetrate, permeabilize, and depolarize the bacterial membrane. Our overall results suggest that peptide μH is an important consideration when designing membrane-active peptide EPIs, as a designed TM with a large μH has the potential to function in an AMP-like manner to induce membrane permeabilization.

## Results

### Peptide design and characterization

Our lab has previously used the natural TM1 sequence from AcrB to synthesize a series of TM peptides designed to stably insert into the bacterial membrane and competitively interfere with the TM1-TM8 oligomerization motif of AcrB to subsequently prevent efflux activity (Fig. 1 and Table 1) (22, 23). To improve solubility and specificity of targeting, these peptides were synthesized with an N-terminal acetyl-A-(Sar)_3_ tag and a C-terminal lysine tag—a design strategy that has been implemented by our lab to target the TM4-TM4 dimerization motif of small multidrug resistance (SMR) efflux pumps from *E. coli*, *Pseudomonas aeruginosa*, and other strains of MDR bacteria (14, 24, 25).

**Table 1:** Sequences of peptides used in this study

Peptide	Sequence^a^	μH^b^	H^b^	MIC^c^ (μM)
TM1-1	Ac-A-(Sar)_3_-PIFAWVIAIIIMLAGGLAILKL-KKKK-NH_2_	0.232	0.832	>64
TM1-2	Ac-A-(Sar)_3_-VPILLIFGAMAGWILIAIIAKL-KKKK-NH_2_	0.265	0.832	>64
TM1-3	Ac-A-(Sar)_3_-VPGLLAFAIMIIAWLGIAIIKL-KKKK-NH_2_	0.297	0.832	>64
TM1-4	Ac-A-(Sar)_3_-VPGLLGFIAMAIWILIAIIAKL-KKKK-NH_2_	0.339	0.832	>64
Melittin	GIGAVLKVLTTGLPALISWIKRKRQQ-NH_2_	-	-	5 ± 2

a Sequence of TM1-1 is based on the helical segment of TM1 from AcrB, while TM1-2, TM1-3, and TM1-4 are reorganized with increasing μH. Ac, acetylated N-terminus; Sar, sarcosine or N-methylglycine; NH_2_, amidated C-terminus.

b Hydrophobic moment (μH) and hydrophobicity (H) measured using the hmoment and hydrophobicity functions of the Peptides R-package (version 2.4.4), respectively.

c MIC determined as peptide concentration resulting in >99% reduction in growth of *E. coli* K12 and are presented as mean and standard deviation of the mean.

**Figure 2:**
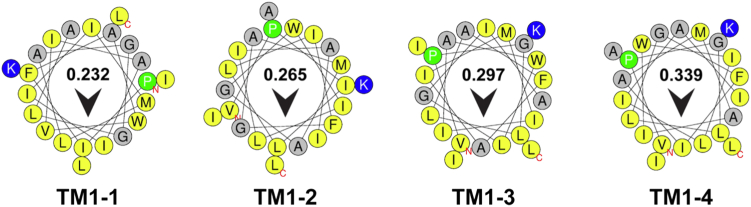
**Helical wheel projections of TM1-based peptidesused in this study.** Hydrophobic amino acids are depicted in *yellow*, and charged amino acids are depicted in *blue*. Small side-chain residues Gly and Ala are depicted in *gray*. The *arrow* and number depict the orientation and value of the hydrophobic moment. N and C represent N- and C-termini, respectively. Helical wheels were generated using the HeliQuest web server (47).

In the present work, we sought to investigate how these peptides were associating with and penetrating the lipid bilayer—a critical step for the peptides’ designed mechanism of action. It became apparent that the μH, *namely*, the degree of localization of hydrophobicity on one face of an α-helical peptide, was contributing to the activity of these inhibitor peptides. Thus, variants of TM1-1 (previously TM1) and TM1-4 (previously TM1_Scr_) (19)—here denoted TM1-2 and TM1-3—were designed with increasing values of μH (Fig. 2 and Table 1). It is noted that all four peptides contain the same amino acid composition, charge, and overall hydrophobicity but their sequences are systematically reorganized to differ only in the value of μH. Despite these differences in μH, none of the four peptides displayed an effective MIC up to 64 μM against *E. coli* K12, whereas the control AMP melittin completely prevents bacterial growth at just 5 μM (Table 1). Subsequent assays were performed with sub-lethal concentrations of the TM1-based peptides to a maximum of 8 μM.

**Figure 3:**
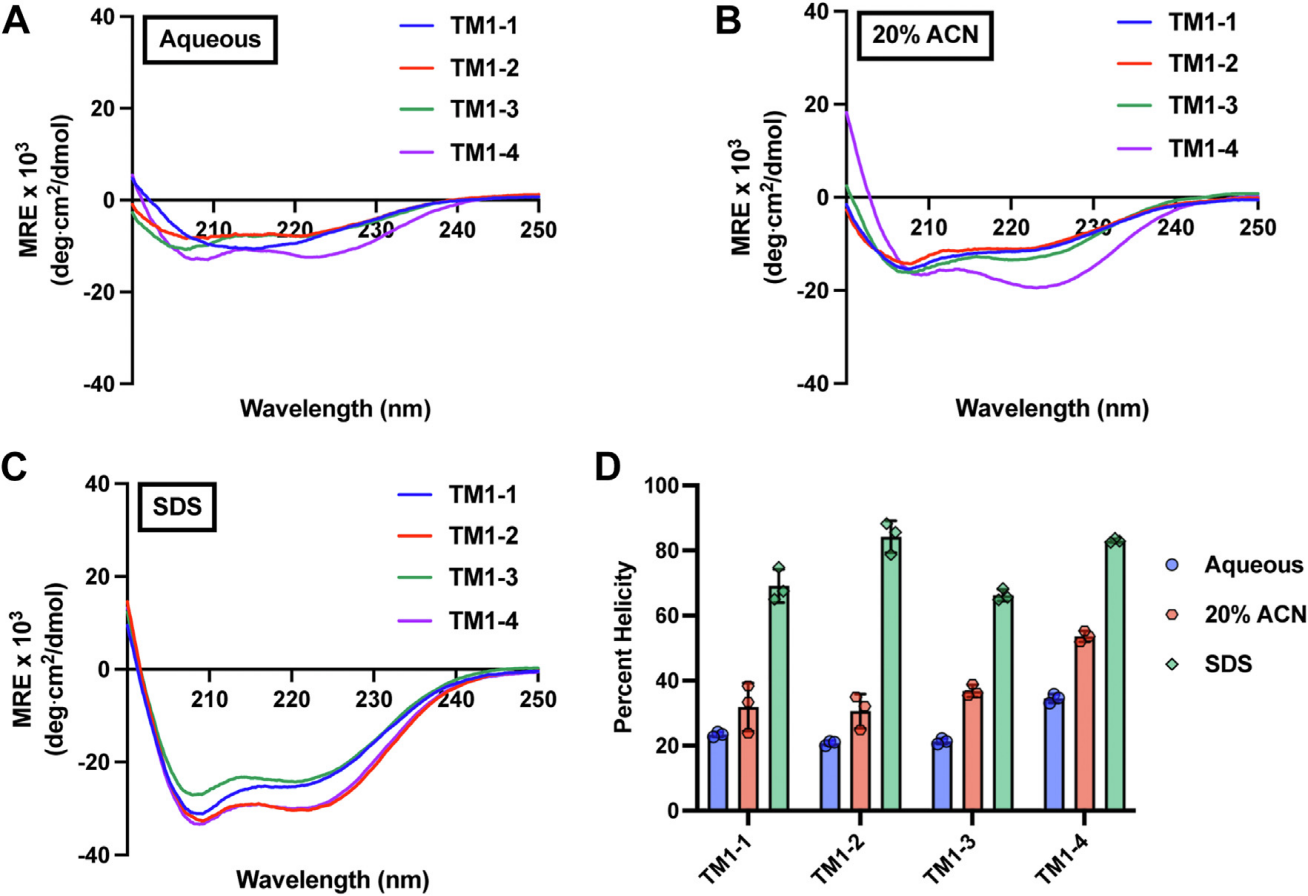
**Peptide secondary structure in various environments.** 20 μM of each peptide was brought up in (*A*) aqueous, (*B*) 20% acetonitrile (ACN), or (*C*) 140 mM SDS, and circular dichroism was performed to assess peptide secondary structure. Each spectrum represents the average of three independent analyses. *D*, Using the mean residue ellipticity (MRE) value at 222 nm, peptide helicity was estimated for each environment. Data are presented as the mean and standard deviation of the mean.

For these peptides to carry out their designed mechanism of action, they must be able to adopt an α-helix and penetrate the lipid bilayer. Circular dichroism (CD) spectroscopy was therefore utilized to assess peptide secondary structure in various environments (Fig. 3). In the aqueous phase, all peptides—excluding TM1-1—adopt weakly α-helical structures, with TM1-4 displaying the strongest helix, indicated by the minima at 208 and 222 nm (Fig. 3*A*). Peptide secondary structure was also assessed in a 20% acetonitrile solution (26), wherein all peptides displayed α-helical structure, although less strongly than in the presence of SDS (Fig. 3, *B* and *C*). Using the ellipticity of these inhibitor peptides at 222 nm, the helical percentage for each peptide in each environment was estimated per Equation 2 (see Experimental procedures) (Fig. 3*D*).

We then used the estimated helical percentage to assess the likelihood of partitioning into the lipid bilayer. Free energy of partitioning to the water-bilayer interface with and without considering peptide helicity (ΔG_IF-Helix_ and ΔG_IF_, respectively) and from the interface to the bilayer (ΔG_OCT-IF_) were estimated with the Totalizer module of MPEx (27). With spontaneous ΔG_IF_ values—regardless of helical consideration—and ΔG_OCT-IF_ ≤20 kcal/mol, we can reasonably conclude these peptides have favorable potential to partition into the lipid bilayer (28) (Table 2).

**Table 2:** Thermodynamic parameters of peptide-membrane association

Peptide	ΔG_IF_^a^ (kcal/mol)	ΔG_IF-Helix_^a^ (kcal/mol)	ΔG_OCT-IF_^a^ (kcal/mol)
TM1-1	−3.01	−6.85	8.80
TM1-2	−3.01	−6.73	8.80
TM1-3	−3.01	−7.45	8.80
TM1-4	−3.01	−9.49	8.80

a Free energy of peptide partitioning was estimated using the Totalizer module of MPEx. ΔG_IF-Helix_, partitioning to the water-bilayer interface without considering peptide helicity; ΔG_IF_, partitioning to the water-bilayer interface considering peptide helicity in 20% acetonitrile; ΔG_OCT-IF_, partitioning from the interface to bilayer.

### Peptide-mediated membrane permeabilization

**Figure 4:**
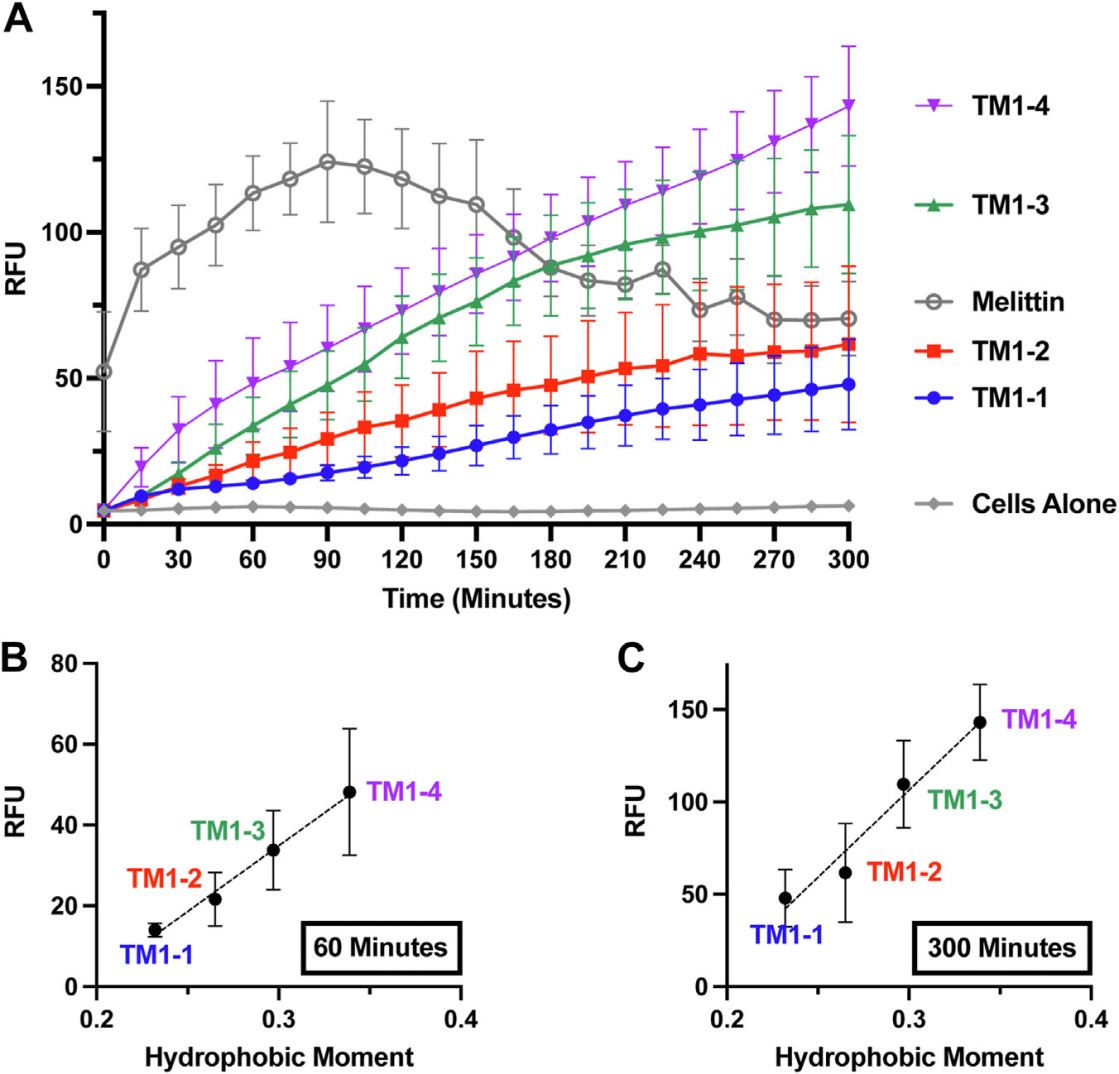
**Membrane permeabilization assay.***A*, *E. coli* K12 cultures were incubated with 5 μM SYTOX for 30 min prior to the addition of 8 μM of each peptide. Fluorescence was measured immediately for up to 300 min. Relative fluorescence units (RFU) at (*B*) 60 min and (*C*) 300 min were plotted against peptide μH. *Dashed line* represents a simple linear regression of the data (b: r^2^ = 0.69, *p* value < 0.0001; c: r^2^ = 0.77, *p* value < 0.0001). Data are presented as mean and standard deviation of the mean. Experiments were performed in duplicate and repeated on four independent days.

While our lab has demonstrated that these peptides are useful at preventing AcrB-mediated efflux activity with minimal liposome disruption *in vitro*, we had yet to determine the peptide-mediated effects on the bacterial membrane in live bacteria (19). To assess these potential effects, we utilized SYTOX Green nucleic acid stain, which has been used extensively as a marker for membrane permeabilization (29, 30, 31). *E. coli* K-12 incubated with SYTOX Green in the absence of peptide was used as a baseline negative control for membrane permeabilization, whereas the pore-forming AMP melittin was used as a positive control (Fig. 4*A*). With a lag time of only a few minutes between peptide addition and fluorescence monitoring, melittin induces immediate membrane permeabilization, indicated by the rapid increase in relative fluorescence units (RFU) (Fig. 4*A*). In comparison, the addition of an equivalent concentration of TM1-based peptides results in a relatively slower increase in RFU, which may be suggestive of the varying affinities for the lipid bilayer. Nonetheless, after 60 min, the peptides displayed varying extents of membrane permeabilization that correlated well with peptide μH, an observation that remained consistent throughout the experiment to the 300-min mark (Fig. 4, *B* and *C*).

### Peptide-induced dissipation of the proton motive force

**Figure 5:**
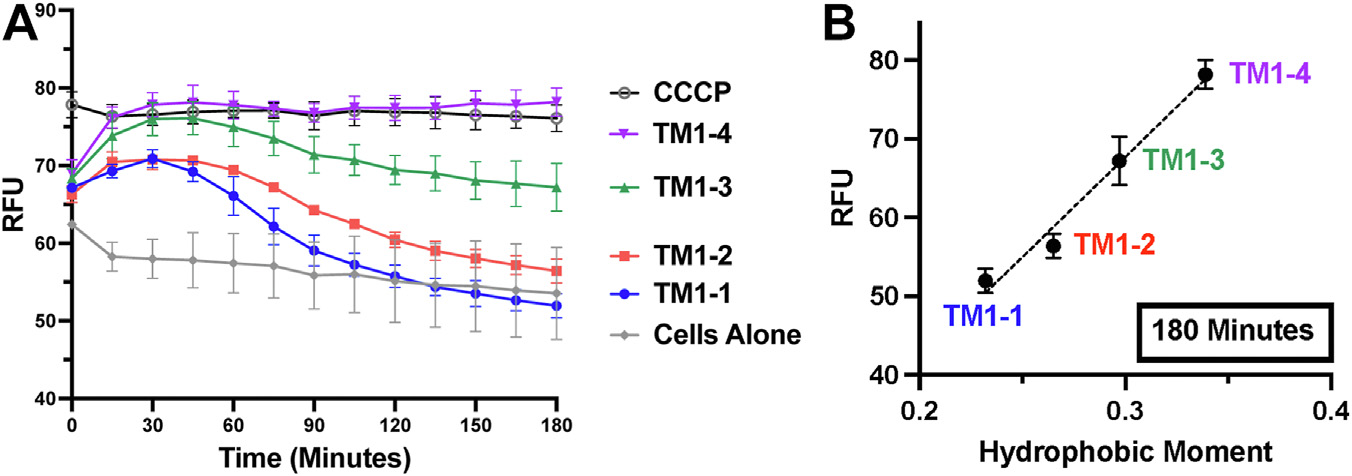
**Proton motive force dissipation assay.***A*, *E. coli* K12 cultures were incubated with 30 μM DiOC_2_ (3) for 5 min prior to the addition of 20 μM CCCP or 8 μM of each peptide. Fluorescence was measured immediately for up to 180 min (*B*) RFU at 180 min were plotted against peptide μH. Dashed line represents a simple linear regression of the data (r^2^ = 0.95, *p* value < 0.0001). Data are presented as mean and standard deviation of the mean. Experiments were performed in duplicate and repeated on three independent days.

With the TM1-based peptides displaying varying levels of bacterial membrane permeabilization, we undertook to assess the impact of permeabilization on the proton motive force (PMF), which is necessary for AcrB-mediated efflux (7, 8). With the fluorescent dye DiOC_2_ (3), we can detect a loss of PMF by an increase in RFU (32, 33). In comparison to untreated *E. coli* K12, the addition of the protonophore CCCP results in an immediate increase in RFU, indicating dissipation of the PMF (Fig. 5*A*). Upon addition of peptide, we see immediate increases in RFU for all peptides; however, cultures incubated with TM1-1 and TM1-2 appear to re-establish the PMF back in the range of untreated cultures (Fig. 5*A*). By the end of the experiment, dissipation of the PMF correlates well with peptide μH (Fig. 5*B*).

### Peptide-LUV association and depth of insertion

**Figure 6:**
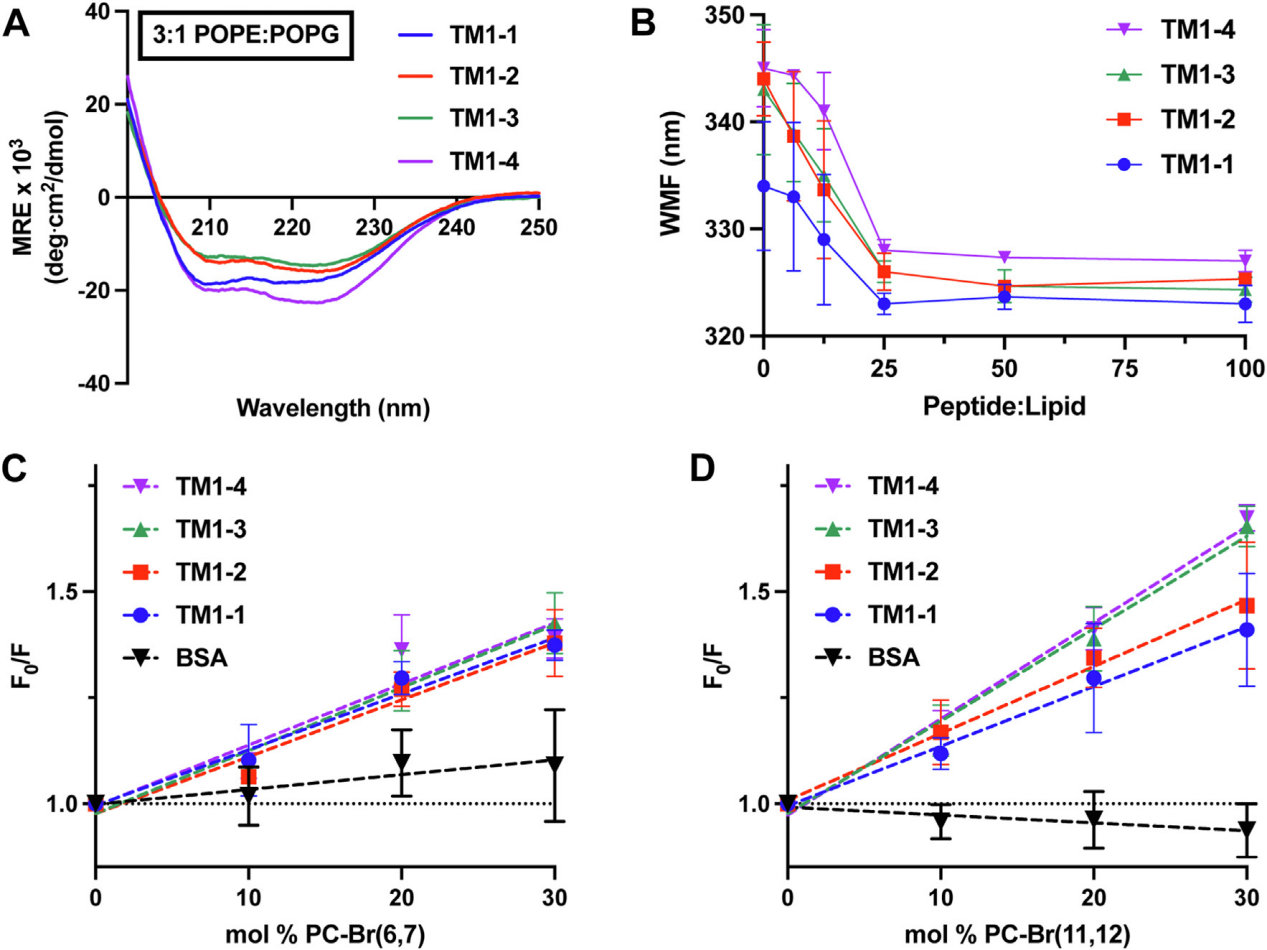
**Peptide-membrane interaction assays.***A*, 20 μM of each peptide was added to LUVs composed of 3:1 POPE:POPG at a peptide-to-lipid ratio of 62.5 and circular dichroism was performed to assess peptide secondary structure. *B*, 10 μM of each peptide was then incubated with LUVs of the same composition at various peptide-to-lipid ratios (0–100) and fluorescence spectra was recorded from 310 to 360 nm to determine the wavelength of maximum fluorescence (WMF). *C*, Stern-Volmer plots of tryptophan fluorescence quenching of 5 μM of each peptide or BSA by PC-Br (6, 7); and (*D*) by PC-Br (11, 12) containing LUVs at a peptide-to-lipid ratio of 62.5. *Dashed line* represents a simple linear regression of the data. Data are presented as the mean of three independent analyses and standard deviation of the mean.

Considering the seemingly μH-driven differences in bacterial membrane permeabilization and depolarization, we sought to investigate the peptide-lipid interaction further using tryptophan fluorescence in bacterial membrane mimetic LUVs. When incubated with LUV’s composed of 3:1 POPE:POPG, peptides adopt typical α-helical structure (Fig. 6*A*). Using the wavelength of maximum tryptophan fluorescence (WMF) as a marker for LUV association, we find that the full TM1-based peptide set has relatively the same affinity for the lipid bilayer, as their WMF all plateau at a peptide-to-lipid ratio of 25 onward (Fig. 6*B*). Despite their similarities in affinity, there may still be differences in the depth each peptide can penetrate the lipid bilayer. To assess this potential difference, we utilized dibrominated lipids, which contain bromine atoms in the six and 7 (PC-Br (6, 7)) or the 11 and 12 (PC-Br (11, 12)) position of the lipid tail and function to quench tryptophan fluorescence based on the depth of peptide insertion (34). As with the CD analysis, peptide was added at a peptide-to-lipid ratio of 62.5, to ensure the results achieved would be at the plateau portion of the WMF curve, when the peptide should be maximally associated with and inserted into the lipid bilayer. Soluble BSA was used as a control for this assay, which shows little to no quenching in the presence of dibrominated PC-containing LUVs (Fig. 6, *C* and *D*). In the presence of PC-Br (6, 7), the TM1-based peptide set shows relatively the same quenching; however, in the presence of PC-Br (11, 12), the peptides with the largest μH—TM1-3 and TM1-4—are able to penetrate deeper into the lipid bilayer, as indicated by the increased quenching slope (Fig. 6, *C* and *D*).

## Discussion

The AcrB TM1-based peptide set used in this work—which consists of four peptides with identical composition, charge, length, and average hydrophobicity, along with the same N- and C-terminal tags—displayed varying degrees of membrane permeabilizing activity up to and beyond the level of the pore-forming AMP melittin (Fig. 4*A*). Importantly, we found that within this TM1-based peptide set, peptide μH correlates well with the propensity to permeabilize the membrane (Fig. 4*B* and *C*). Further, when we assessed the consequences of permeabilization on membrane potential, we found that peptide μH also correlates well with dissipation of the PMF, indicating the peptides may be contributing, in part, to preventing AcrB-mediated efflux via this mechanism as well (Fig. 5*B*). While a study of amphipathic arginine-rich AMPs revealed a similar correlation between peptide μH and general membrane perturbation (35), the present work is to our knowledge the first observation of such correlation with the peptide μH of lipopathic TMs.

Interestingly, addition of each TM1-based peptide results in an initial dissipation of the PMF around the 30-min mark, which may indicate that peptide penetration of the membrane occurs within that time frame (Fig. 5*A*). However, in comparison to the activity of the AMP melittin—used here as a positive control for membrane permeabilization—the TM1-based peptides resulted in a much slower increase in RFU, suggesting an inherent difference in the mechanism by which these peptides associate with or penetrate the bacterial membrane (Fig. 4*A*). We suspect this may be attributable to a necessity for lipid recruitment by the TM1-based peptides. Our lab has previously proposed a model of lateral-phase separation, in which two distinct “pools” of lipids are formed upon the addition of TMs, an observation that has been noted in the context of LL-37-derived AMP fragments (14, 36). Given the charge-based interaction by which our peptides are designed, we can infer the peptides would recruit negatively charged POPG into POPG-enriched and POPG-depleted lipid pools. This necessity for lipid recruitment may also explain why the initial dissipation of the PMF by TM1-based peptides takes 30 min to achieve, whereas CCCP displays immediate dissipation (Fig. 5*A*).

With strong evidence that peptide μH may be driving bacterial membrane permeabilization and depolarization by TMs, we investigated this further in a controlled *in vitro* system using 3:1 POPE:POPG LUVs—the typical lipid mixture for modeling bacterial inner membranes (37, 38). Dibrominated lipids were utilized to assess the depth at which these peptides are penetrating the lipid bilayer, with the results revealing the level at which peptide μH may be a driving factor. Each of the peptides is quenched to a similar degree with the PC-Br (6, 7) lipids; however, the peptides with the largest μH—TM1-3 and TM1-4—are quenched to a higher degree with the PC-Br (11, 12), indicating they may be penetrating the LUVs closer to the core (Fig. 6, *C* and *D*). The observed non-linear increase in quenching as a function of peptide μH may suggest there is a threshold of μH—likely between the μH of TM1-2 and TM1-3 for this set of peptides—that drives deeper penetration by TMs, similar to the threshold of overall hydrophobicity that can dictate peptide association with the lipid bilayer (39, 40, 41).

**Figure 7:**
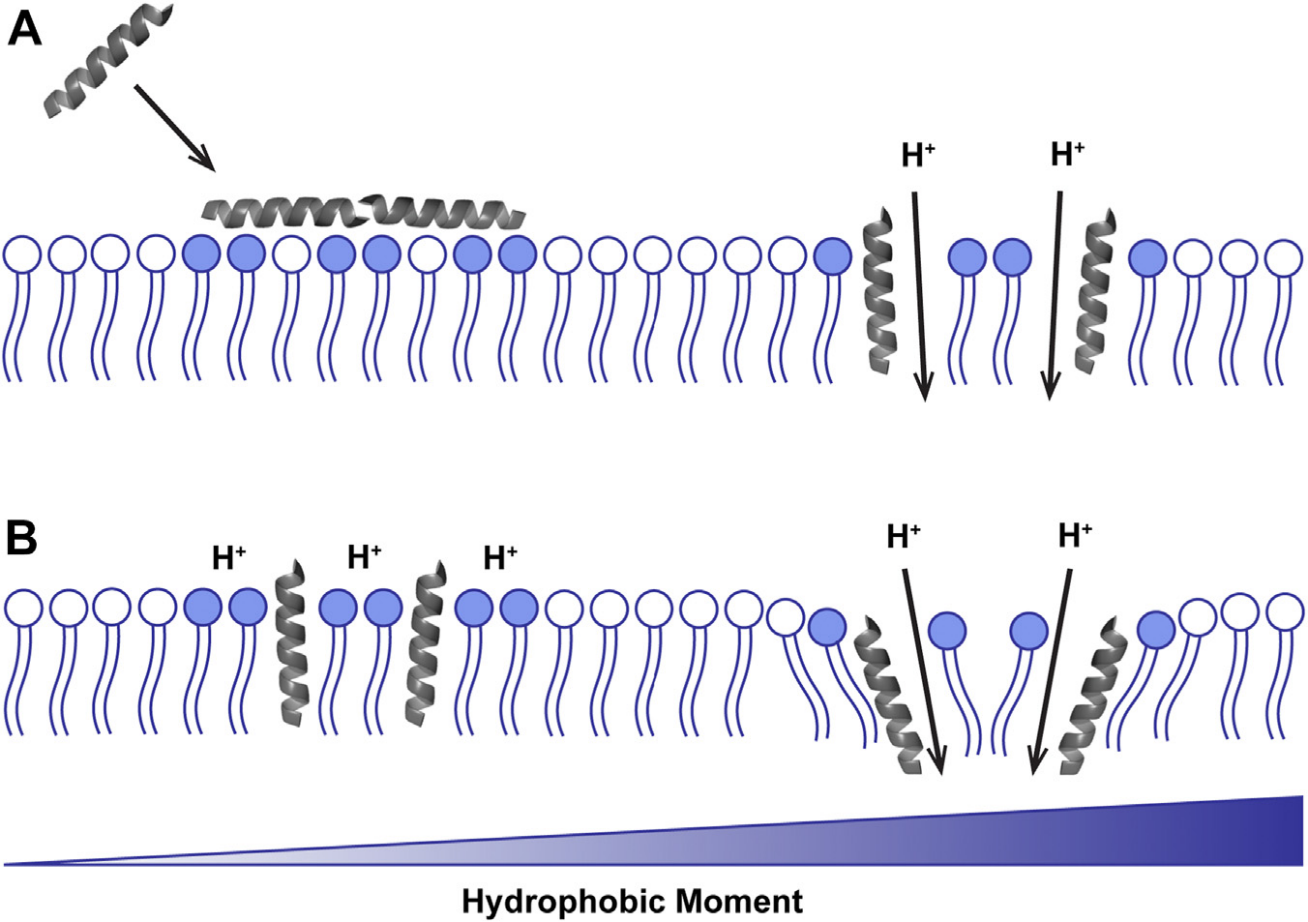
**Proposed model of peptide association with and penetration into lipid bilayers.***A*, Peptides are attracted to the bacterial membrane through electrostatic forces with the negatively charged POPG (filled in phospholipid heads). Peptide adsorption recruits POPG (*left*) and allows the peptide to penetrate the bacterial membrane, causing an initial dissipation of the PMF (*right*). *B*, Peptides may penetrate the bacterial membrane deeper depending on the value of their μH. Peptides with a smaller μH will remain closer to the surface of the bilayer, allowing the PMF to re-establish (*left*), whereas peptides with a larger μH will penetrate closer to the core of the bilayer, further contributing to membrane permeabilization and dissipation of the PMF (*right*).

The present findings lead to the model for TM peptide association and penetration into bacterial membranes shown schematically in Figure 7. Upon adsorption of the cationic TM peptide to the bacterial membrane via electrostatic interactions with negatively charged POPG, there is an initial lipid recruitment phase that allows these peptides to penetrate the lipid bilayer. Accompanying this insertion event, there is a dissipation of the PMF that can be coupled with membrane permeabilization. Once embedded in the bacterial membrane, the peptide μH then contributes to the depth to which these peptides can penetrate, subsequently destabilizing the packing of lipids further to maintain the dissipation of the PMF.

It remains of interest to inquire whether one can identify an optimal μH within the present peptide set that simultaneously allows maximal membrane penetration without compromising PPI-specific activity. Such a possibility raises the broader question as to whether a universal, optimal μH exists for any membrane-active peptide. However, it is likely that additional factors—such as peptide length and charge, as well as any target PPIs—would prevent discerning a universal μH that would be applicable to all peptides. We therefore suggest that an optimal μH may only exist within a given peptide set, which will determine the impacts on the bacterial membrane upon penetration. Modification of this optimal μH can allow the user to introduce new functions, such as membrane permeabilization and depolarization, into their peptide design according to the utility of the peptide.

## Conclusion

In TM peptides that have predominantly hydrophobic sequences, the present work has shown that the peptide face with the largest μH may preferentially orient itself towards the most hydrophobic region of the lipid bilayer (*i.e.*, the core vs. the surface). This observation, in turn, provides specific peptide design strategies to guide the depth and orientation through which TM peptides associate with lipid bilayers. As such, by simultaneously targeting membrane-embedded PPIs, while inducing membrane permeabilization and depolarization without toxicity, our overall results suggest that TMs with appropriately chosen μH’s can function as EPIs via multiple mechanisms to treat clinically relevant MDR bacterial infections.

## Experimental procedures

### Peptide design and quantification

Peptides used in this study were purchased from Biosynth (Lelystad, NL, Louisville, KY, Gardner, MA, USA) with >90% purity. Peptides were shipped as lyophilized powder and were solubilized in either 2,2,2-trifluoroethanol or 20% acetonitrile for quantification. As each peptide contains a single tryptophan residue, peptide concentration was assessed by the absorption at 280 nm using a 1 cm pathlength quartz cuvette and an Ultrospec 3000 UV/Vis spectrophotometer (Pharmacia Biotech). Peptides were subsequently stored as lyophilized powder at −20 °C or solubilized in DMSO for experimental use. Melittin control peptide was purchased from Sigma Aldrich (St Louis, MO, USA) and solubilized in DMSO for experimental use. Peptide hydrophobic moment (μH) and hydrophobicity (H) were calculated using the R-package Peptides (version 2.4.4) (42), which utilizes the Eisenberg hydrophobicity scale (43, 44). Lysine tags were omitted from these calculations, as the tag is designed to direct the peptide towards, but not penetrate, the bacterial membrane.

### Minimum inhibitory concentration assay

Overnight cultures of *E. coli* K-12 grown in Mueller-Hinton broth were incubated at 50,000 CFU/well with up to 64 μM of peptide in 96-well plates (Corning, Ref 3598). Plates were then incubated at 37 °C for 20 h, after which the optical density (OD) was measured at 600 nm. Minimum inhibitory concentration (MIC) was determined as the peptide concentration resulting in >99% reduction in growth.

### Circular dichroism and helical quantification

Circular dichroism (CD) spectra of peptides were recorded between 190 and 250 nm on a Jasco J-1500 CD spectropolarimeter using a 0.1 cm pathlength quartz cuvette, scanning at 50 nm/s with three accumulations. Peptide spectra were acquired in either water, 20% acetonitrile, 140 mM sodium dodecyl sulfate (SDS), or 3:1 POPE:POPG, in buffer (10 mM Tris-Cl, 10 mM NaCl, pH 7), at a constant peptide concentration of 20 μM and then plotted using GraphPad Prism 9. Data are presented from 200 to 250 nm for ease of visualization. Raw millidegree (mdeg) spectra were background subtracted and converted to mean residue ellipticity (MRE) using Equation 1 below, where *c* is the concentration in μM, *l* is the pathlength in cm, and *n* is the number of residues.

(1) $$MRE=\frac{100\left(mdeg\right)}{cln}$$

Using the MRE value at 222 nm, the peptide helical percentage was estimated using Equation 2 below (45, 46), where *MRE*_*222*_ is the MRE value at 222 nm and *n* is the number of peptide bonds.

(2) $$\%\;Helix=100\;\left(MRE_{222}/\left(-39,500\;\left(1-2.57/n\right)\right)\right)$$

### Thermodynamic assessment via MPEx

Free energy of peptide partitioning to the water-bilayer interface without considering peptide helicity (ΔG_IF_) and with considering peptide helicity (ΔG_IF-Helix_), as well as from the interface to bilayer (ΔG_OCT-IF_), were estimated using the Totalizer module of Membrane Protein Explorer (MPEx) software (27) (https://blanco.biomol.uci.edu/mpex/). To stay as true to peptide design as possible, sarcosine residues were substituted for alanine, and N-terminal acetylation and C-terminal amidation were included in the analysis.

### Membrane permeabilization assay

Membrane permeabilization was assessed by adapting methods previously described, with modifications (30). Overnight cultures of *E. coli* K-12 grown in LB broth were pelleted as described and resuspended to an OD_600_ of 0.2 in either M9 minimal media or 70% isopropanol to achieve viable and permeabilized cells, respectively. Cultures were incubated for 30 min prior to being pelleted and resuspended in fresh M9 minimal media. SYTOX Green Nucleic Acid Stain (Invitrogen) was added to cultures to a final concentration of 5 μM and incubated in the dark for 30 min. Black, opaque 96-well plates (Corning, Ref 3650) were filled with 20 μl water per well containing 8 μM of the respective peptide. 80 μl of SYTOX-containing culture were added to each well and fluorescence was measured immediately at 37 °C every 3 min for up to 5 h using a SpectraMax Gemini EM microplate reader set to 488 nm excitation and 523 nm emission wavelengths. Fluorescence curves were normalized to isopropanol-treated cultures and then plotted and analyzed using GraphPad Prism 9. Data at 15-min intervals are presented as mean and standard deviation of the mean for ease of visualization. Experiments were performed in duplicate and repeated on four independent days.

### Proton motive force dissipation assay

Dissipation of the proton motive force (PMF) was assessed by adapting methods previously described, with modifications (32). Overnight cultures of *E. coli* K12 grown in Luria-Bertani (LB) broth were pelleted and resuspended to an OD_600_ of 0.4 in M9 minimal media. Cultures were grown at 37 °C for 30 min prior to being incubated with 10 mM EDTA for 5 min. Cultures were pelleted again and resuspended in fresh M9 minimal media to remove EDTA before the addition of 30 μM 3,3′-diethyloxacarbocynanine iodide (DiOC_2_ (3); Invitrogen). Cultures were incubated in the dark for 5 min prior to being added to black, opaque 96-well plates (Corning, Ref 3650) containing 20 μM carbonyl cyanide *m*-chlorophenylhydrazine (CCCP) or 8 μM of the respective peptide. Fluorescence was measured immediately at 37 °C every 3 min for up to 3 h using a SpectraMax Gemini EM microplate reader set to 450 nm excitation and 510 nm emission wavelengths. Fluorescence curves were normalized to wells containing only dye and then plotted and analyzed using GraphPad Prism 9. Data at 15-min intervals are presented as mean and standard deviation of the mean for ease of visualization. Experiments were performed in duplicate and repeated on three independent days.

### LUV preparation

Large unilamellar vesicles (LUVs) were prepared using methods previously described (14). Briefly, 1-palmitoyl-2-oleoyl-sn-glycero-3-phosphoethanolamine (POPE) and 1-palmitoyl-2-oleoyl-sn-glycero-3-phospho-(1′-rac-glycerol) (POPG) (Avanti Polar Lipids) were combined in a 3:1 ratio, respectively, in chloroform and dried to a film before water washing and lyophilizing into a powder. This powder was resuspended in Tris-Cl buffer (10 mM Tris, 10 mM NaCl, pH 7), freeze-thawed 5× (dry ice – 50 °C water bath) and extruded through a mini-extruder fitted with a 0.2 μm Whatman Nuclepore polycarbonate membrane filter (Cytiva) at least 15× to form LUVs. Where applicable, 1-palmitoyl-2-(6,7-dibromo)stearoyl-sn-glycero-3-phosphocholine (PC-Br (6, 7)) or 1-palmitoyl-2-(11,12-dibromo)stearoyl-sn-glycero-3-phosphocholine (PC-Br (11, 12)) (Avanti Polar Lipids) were substituted for POPE at the given percentages and incorporated into LUVs as described.

### Wavelength of maximum fluorescence assay

Prepared LUVs were combined with 10 μM peptide at varying peptide-to-lipid ratios indicated. Tryptophan emission fluorescence was recorded on a Photon Technology International fluorimeter from 310 to 360 nm using a 280 nm excitation wavelength. Fluorescence spectra were background subtracted, plotted, and the wavelength of maximum fluorescence (WMF) was measured using the area under the curve method via GraphPad Prism 9. Data are presented as the mean of three independent analyses and standard deviation of the mean.

### Tryptophan quenching assay

Brominated lipid-containing LUVs (0%, 10%, 20%, and 30%) were combined with 5 μM peptide and tryptophan emission fluorescence was recorded from 300 to 400 nm using a 280 nm excitation wavelength. Fluorescence spectra were plotted and the area under the curve was used to generate a Stern-Volmer plot of initial fluorescence/quenched fluorescence (F_o_/F) against percentage brominated lipid. Bovine serum albumin (BSA; Sigma-Aldrich) was used at the same concentration as peptide as a control for quenching. Data are presented as the mean of three independent analyses and standard deviation of the mean.

## Data availability

All data are contained within the manuscript.

## Conflict of interest

The authors declare that they have no conflicts of interest with the contents of this article.
